# Hippocampal Volume Mediates the Association Between Serum Ferritin and Cognition in Non‐Dialysis End‐Stage Renal Disease Patients

**DOI:** 10.1002/brb3.71777

**Published:** 2026-09-17

**Authors:** Jingjing Si, Minghua Zhu, Mingzhe Dai

**Affiliations:** ^1^ Department of Nephropathy The First Affiliated Hospital of Anhui Medical University Hefei Anhui China

**Keywords:** cerebral atrophy, cognitive impairment, end‐stage renal disease, magnetic resonance imaging

## Abstract

**Objective:**

Quantitatively assess the characteristics of brain volume in end‐stage renal disease (ESRD) individuals through high‐resolution structural MRI, analyze the relationship between these features and cognitive impairment, as well as explore associated risk factors.

**Methods:**

Fifty ESRD subjects and 48 healthy controls, matched by demographics, participated in the study. Each participant underwent neuropsychological assessments and 3.0T MRI scans, with cerebral volume data obtained via voxel‐based morphometry.

**Results:**

ESRD patients showed significantly lower scores in mini‐mental state examination (MMSE) and Montreal cognitive assessment (MoCA), along with reduced gray matter volume (GMV) that was positively correlated with cognitive scores. After adjusting for confounding factors, serum ferritin levels independently served as a risk factor contributing to the decrease in GMV, with hippocampal volume partially mediating the ferritin‐cognition association.

**Conclusions:**

Lower serum ferritin levels may exert a protective effect against the decrease in gray matter volume and the deterioration of cognitive function in non‐dialysis ESRD individuals.

## Graphical abstract

Cognitive impairment is a prevalent neurological complication of end‐stage renal disease (ESRD), yet the neuroanatomical pathway linking iron overload to cognitive decline remains unclear. This study enrolled 50 nondialysis ESRD patients and 48 demographically matched healthy controls, all of whom underwent 3.0T structural MRI for voxel‐based morphometry (VBM)‐based gray matter volume (GMV) quantification and standardized cognitive assessments (MMSE, MoCA). Correlation analysis and bootstrap mediation modeling revealed that nondialysis ESRD patients had significantly reduced global and regional GMV accompanied by cognitive decline; elevated serum ferritin was an independent risk factor for GMV reduction, and hippocampal volume played a significant partial mediating role in the ferritin‐cognition association. These findings provide novel neuroimaging evidence for iron‐related cognitive damage in ESRD, and support the optimization of clinical iron management to protect neurological function.

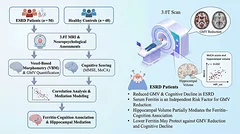

## 1 Introduction

Chronic kidney disease (CKD) refers to a continuous and progressive decline in kidney function, indicated by the presence of albumin in urine or a glomerular filtration rate (GFR) under 60 mL/min/1.73m^2^ for a duration exceeding 3 consecutive months. As CKD advances, a further reduction in GFR to levels of 30 or 15 mL/min/1.73m^2^ designates the progression to advanced stages of the disease and end‐stage renal disease (ESRD), respectively (Kalantar‐Zadeh et al. 2021). The global prevalence of CKD exceeded 700 million in 2017, with related deaths reaching 1.2 million; it is projected that CKD‐associated mortality will surge to 2.2 million to 4 million by 2040 (GBD Chronic Kidney Disease Collaboration 2020). Individuals suffering from ESRD face not only risks of complications (cardiovascular events, infections, and cerebrovascular accidents), but there is also growing concern regarding neurological and psychiatric involvement, especially cognitive impairment, which has attracted increasing attention recently. Emerging neuroimaging research has demonstrated that patients with ESRD generally exhibit extensive cerebral structural alterations, which may serve as the neuroanatomical basis for their cognitive decline, elevated stroke risk, and impaired quality of life (Wang et al. 2024; Gupta et al. 2016).

A large body of research data based on structural magnetic resonance imaging (sMRI) has indicated that CKD may be associated with cerebral pathologies including white matter lesions, cerebral infarction, and cerebral atrophy (Gupta et al. 2016; Drew et al. 2013). Patients with ESRD showed significant gray matter volume (GMV) reductions in regions of the limbic system and default mode network (Zhang et al. 2013). In contrast, the decrease in white matter volume often reflects microstructural damage such as axonal integrity disruption and demyelination (Scaravilli et al. 2024). However, the findings of existing studies remain inconsistent. Meanwhile, several factors may potentially exacerbate cerebral structural damage in ESRD patients, including uremic toxin accumulation, blood pressure fluctuations, calcium–phosphorus metabolism disorders, secondary hyperparathyroidism, chronic inflammatory state, and anemia (Bossola and Picconi 2024; Hamed 2019). Clarifying the associations between these clinical variables and cerebral structural alterations is of crucial significance for identifying high‐risk patients, elucidating the underlying injury mechanisms, and developing targeted neuroprotective strategies. Consequently, this study seeks to systematically assess differences in cerebral structure among ESRD patients using high‐resolution structural magnetic resonance imaging, and further explore the correlations between cerebral structural metrics and key clinical as well as laboratory indicators of the patients, ultimately offering potential targets for the early detection and intervention of neurocognitive complications.

## 2 Materials and Methods

### 2.1 Study Population

ESRD individuals (CKD Stage 5) who had not undergone any form of renal replacement therapy and participated in clinical assessments, laboratory evaluations, and comprehensive neuroimaging examinations in the Department of Nephrology, the First Affiliated Hospital of Anhui Medical University, from June 2024 to March 2025 were enrolled in this study, with the following inclusion criteria: (1) meeting the diagnostic criteria for Stage 5 chronic kidney disease (CKD 5) established by the National Kidney Foundation (NKF); (2) complete clinical data available; and (3) aged 18–80 years old. Individuals were not included if they fulfilled any of the subsequent conditions: (1) a history of alcohol dependence, traumatic brain injury, intracranial space‐occupying lesions, Parkinson's disease, epilepsy, dementia, major depressive disorder, or other neurological diseases; (2) visual or auditory impairment, or other severe systemic diseases including malignant tumors, cardiac insufficiency, severe hepatic dysfunction, or autoimmune diseases; and (3) severe claustrophobia or contraindications to magnetic resonance imaging (MRI). The calculation of the sample size utilized the formula applicable to cross‐sectional studies: *n* = (Zα^2^ × *P* × (1 − *P*)) / *d* ^2^. With an allowable error margin set at 20%, *α* = 0.05, and an estimated population proportion (P) of approximately 70%, the necessary sample size amounted to 42 cases. In addition, a total of 50 healthy controls (HCs) were selected from the local community, ensuring they were comparable to the patients regarding gender, age, and educational background. Out of these, 2 individuals were excluded because of cerebral infarction or claustrophobia. This cross‐sectional research received approval from the Research Ethics Committee at the First Affiliated Hospital of Anhui Medical University (Ethics approval number: PJ2024‐06‐63). All participants provided written informed consent, adhering to the Declaration of Helsinki.

### 2.2 Clinical and Biochemical Assessments

Demographic and clinical data of all enrolled patients were collected, including age, gender, body mass index (BMI), smoking and drinking history, iron supplementation history, comorbidities (e.g., diabetes mellitus), and blood pressure. Laboratory‐related indicators: Blood leukocyte count, hemoglobin, creatinine, blood uric acid, serum albumin, lactate dehydrogenase (LDH), alkaline phosphatase, ferritin, transferrin saturation, total cholesterol, triglycerides, serum calcium, serum phosphorus, blood bicarbonate, and parathyroid hormone of the enrolled patients were collected. All the above indicators were measured on an automatic chemistry analyzer using standard laboratory methods. In the HC group, no biochemical blood tests were conducted.

All subjects underwent neuropsychological tests by two senior researchers within 1 h before and after the MRI examination. The evaluation of cognitive abilities was thoroughly conducted through the Montreal cognitive assessment (MoCA) and mini‐mental state examination (MMSE). These assessments encompass various domains, including attention, executive functioning, memory, language and speech capabilities, as well as visuospatial skills, and are used to assess global cognitive function. A MoCA score below 26 indicates mild cognitive impairment (MCI), with lower scores reflecting worse cognitive function (Marcos et al. 2016).

### 2.3 MRI Data Acquisition and Processing

Images obtained through MRI for every participant were captured using a 3.0T MRI system (Siemens Healthcare, Erlangen, Germany) that features a 64‐channel head matrix coil. The main scanning parameters were as follows: T2‐fluid‐attenuated inversion recovery (T2‐FLAIR): echo time (TE) = 98 ms, repetition time (TR) = 8000 ms, field of view (FOV) = 220 mm × 200 mm, flip angle = 150°, matrix = 320 × 224, slice thickness = 5 mm, slice gap = 1 mm, number of slices = 24, and scanning time = 98 s; 3D‐T1 (structural image): TE = 2.9 ms, TR = 2300 ms, FOV = 256 mm × 240 mm, flip angle = 9°, matrix = 256 × 256, slice thickness = 1 mm, slice gap = 0.5 mm, number of slices = 208, and scanning time = 5.1 min.

Subsequent data processing steps were completed using Statistical Parametric Mapping 12 (SPM12, http://www.fil.ion.ucl.ac.uk/spm/) based on MATLAB 2017a (MathWorks, Natick, MA, USA). The structural MRI data underwent preprocessing through the voxel‐based morphometry (VBM) module within the computational anatomy toolbox (CAT12, version r2577). Three‐dimensional T1‐weighted images were subjected to spatial normalization and tissue segmentation, resulting in probabilistic maps that represent GMV, white matter volume (WMV), and total intracranial volume (TIV) compartments. Tissue‐specific volumetric measurements were quantified using the SPM12 software. The automated anatomical labeling atlas (AAL) was nonlinearly coregistered to the individual's native space via a high‐dimensional warping algorithm (DARTEL registration in CAT12), followed by resampling to match the voxel of the preprocessed GM density maps. ROI analyses were conducted using the CAT12 ROI toolbox to extract mean GM volume values across all 116 parcellated regions. Four cognition‐related brain regions (prefrontal cortex, hippocampus, thalamus, and anterior cingulate cortex) were selected as our regions of interest (ROIs) for region‐specific analysis, guided by prior research findings (Rolls 2022; Shine et al. 2023; Alizadeh Mansouri et al. 2024).

### 2.4 Statistical Analysis

SPSS 25.0 and R software (version 4.2.0) were utilized for data analysis. Normal data with homogeneous variance were expressed as mean ± standard deviation and compared using the *t*‐test. Otherwise, data were represented as M (P25, P75) and compared utilizing the Mann–Whitney *U* test. Categorical data were presented in terms of frequency and percentage, with pairwise comparisons performed by the chi‐square test. Correlation analysis employed Pearson or Spearman correlation coefficients. To investigate possible non‐linear relationships between MoCA scores and continuous clinical variables, we performed Spearman's rank correlation analyses and supplemented these with locally weighted scatterplot smoothing (LOWESS) regression curves. The LOWESS curves were fitted with a default span of 0.75 and were overlaid on scatterplots to visually assess the shape of the associations between MoCA scores and key variables.

To conduct a dose‐response relationship assessment between GMV and cognitive function while adjusting for possible confounding factors that could influence cognitive abilities, a restricted cubic spline (RCS) regression was performed. This analysis utilized four knots placed at the fifth, 35th, 65th, and 95th percentiles of the GMV distribution. The overall significance of the spline variables and the nonlinear component were tested using analysis of variance (ANOVA). To investigate if regional GMV mediates the relationship between serum ferritin levels and cognitive function, a causal mediation analysis was performed using the “mediation” package in R. The average causal mediation effect (ACME), average direct effect (ADE), and total effect were calculated using quasi‐Bayesian Monte Carlo simulations, which included 5000 bootstrap iterations. Finally, brain GMV was used as the dependent variable in a multiple linear regression analysis to explore correlations between brain structure and clinical indicators. Variables with *p* values < 0.05 in univariate analysis and those demonstrating clinical significance were included in the multivariate analysis. A two‐tailed *p* < 0.05 was considered statistically significant.

## 3 Results

### 3.1 Demographic Characteristics

**FIGURE 1:**
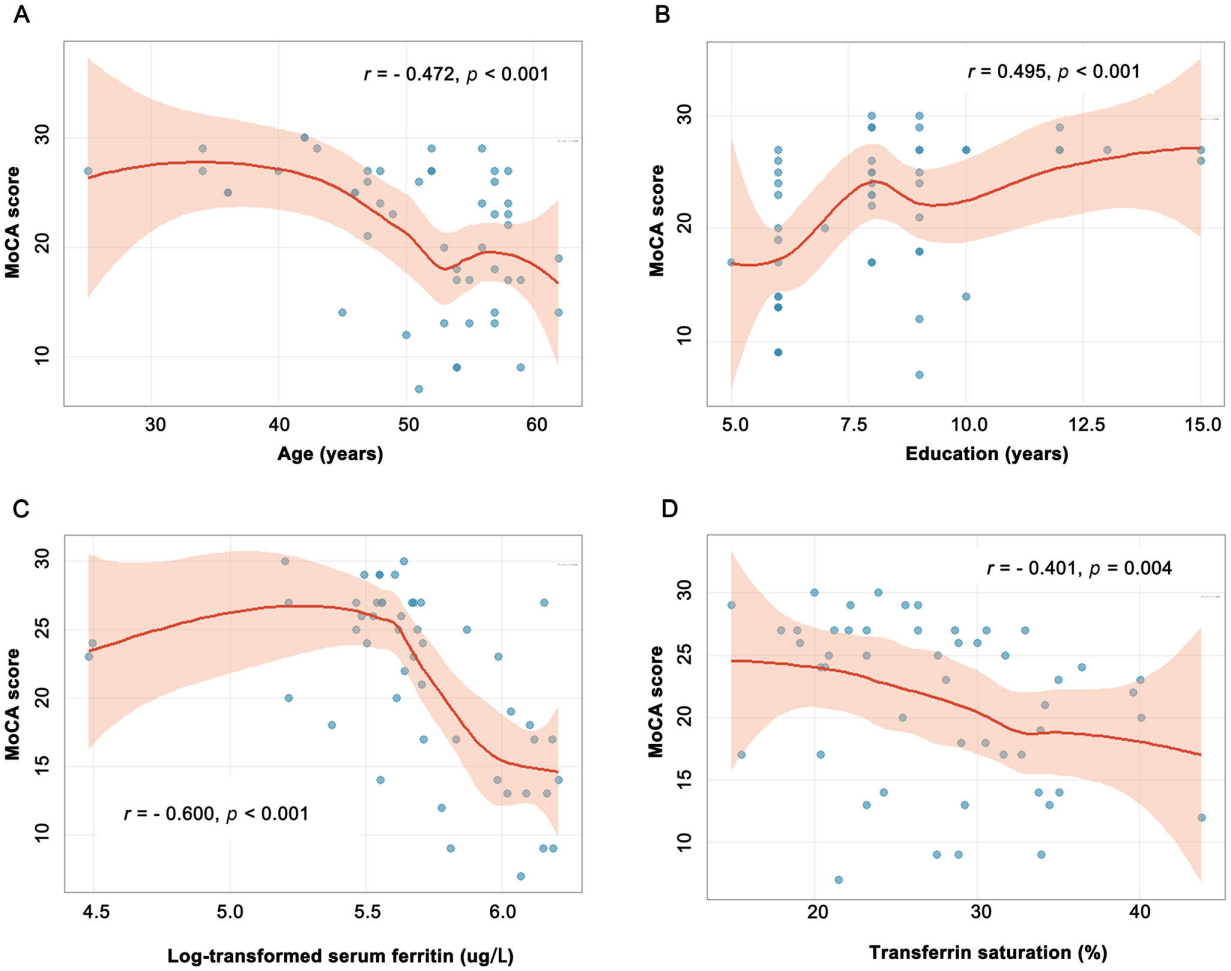
Associations between MoCA scores and clinical variables in nondialysis ESRD patients (*n* = 50). Scatter plots overlaid with locally weighted scatterplot smoothing (LOWESS) curves demonstrate the relationships between Montreal cognitive assessment (MoCA) scores and (A) age, (B) years of education, (C) log‐transformed serum ferritin, and (D) transferrin saturation. Statistical significance was assessed via Spearman rank correlation analysis. (A) *r* = −0.472, *p* < 0.001; (B) *r* = 0.495, *p* < 0.001; (C) *r* = −0.600, *p* < 0.001; and (D) *r* = −0.401, *p* = 0.004. The LOWESS curve in panel D visually reveals a threshold pattern: MoCA scores remain stable at lower ferritin levels and decline sharply when ferritin exceeds approximately 300.4 µg/L. ESRD, end‐stage renal disease; LOWESS, locally weighted scatterplot smoothing; MoCA, Montreal cognitive assessment.

Throughout the duration of the study, our center admitted 62 ESRD patients. Four individuals who had experienced cerebral infarctions, 3 with a background of cerebral hemorrhage, 3 with malignant tumors, and 2 with insufficient clinical data, were excluded sequentially, resulting in a total of 50 patients being enrolled for an MRI examination. The median age was 53 years, including 31 females (62%), with a mean education duration of 8 years. Among the subjects, 11 had a smoking history (22%), 10 had a drinking history (20%), and 9 were complicated with diabetes mellitus (18%). Table 1 presents the relevant details. When comparing the non‐cognitive impairment (NCI) group to the MCI group, the latter exhibited notably higher levels of age, serum ferritin, and transferrin saturation. In contrast, their educational level and diastolic blood pressure were significantly lower. Other variables, such as gender, BMI, and CRP, showed no significant differences between the two subgroups (all *p* > 0.05). As shown in Figure 1A–D, MoCA score was negatively correlated with age (*r* = −0.472, *p* < 0.001), log‐transformed serum ferritin (*r* = −0.600, *p* < 0.001), transferrin saturation level (*r* = −0.401, *p* = 0.004). Conversely, it was positively correlated with educational level (*r* = 0.495, *p* < 0.001). Notably, the LOWESS curve for log‑transformed serum ferritin versus MoCA score revealed a distinctive non‑linear pattern: MoCA scores remained relatively stable at lower ferritin levels, but exhibited a marked and sustained decline once serum ferritin exceeded approximately 300.4 µg/L.​

**TABLE 1:** Demographic and laboratory parameters of nondialysis ESRD patients by cognitive status.

Variables	All (*n* = 50)	NCI (*n* = 18)	MCI (n = 32)	χ^2^/Z/t	*p*
Age (years)	53 (47, 57)	48 (42, 52)	55 (50, 58)	−2.714	0.007
Female [n (%)]	31 (62.0)	12 (66.7)	19 (59.4)	0.260	0.610
Education (years)	8 (6, 9)	9 (8, 12)	8 (6, 9)	−3.601	< 0.001
BMI (kg/m2)	23.17 ± 2.91	23.27 ± 3.06	23.34 ± 2.81	−0.072	0.943
Smoking history [n (%)]	11 (22.0)	5 (27.8)	6 (18.8)	—	0.494
Drinking history [n (%)]	10 (20.0)	5 (27.8)	5 (15.6)	—	0.463
Iron supplementation history [n (%)]	28 (56.0)	10 (55.6)	18 (56.3)	0.002	0.962
Diabetes [n (%)]	9 (18.0)	3 (16.7)	6 (18.8)	—	1.000
Systolic blood pressure (mmHg)	142.12 ± 16.87	145.72 ± 15.65	140.09 ± 17.42	1.136	0.262
Diastolic blood pressure (mmHg)	91.10 ± 12.05	96.06 ± 10.87	88.31 ± 11.93	2.272	0.028
Laboratory data					
White blood cell count (10^9^/L)	6.82 ± 2.30	6.58 ± 2.38	6.95 ± 2.28	−0.540	0.592
Hemoglobin (g/L)	100.72 ± 17.98	99.33 ± 16.72	101.50 ± 18.86	−0.406	0.687
Albumin (g/L)	37.96 ± 3.37	37.57 ± 3.14	38.17 ± 3.53	−0.603	0.550
Alkaline phosphatase (U/L)	84.70 (62.48, 108.23)	85.95 (69.60, 123.10)	84.70 (61.75, 104.45)	−0.505	0.613
LDH (U/L)	227.45 (189.93, 262.65)	210.85 (187.30, 251.30)	236.00 (202.65, 266.05)	−1.152	0.249
Serum creatinine (umol/L)	971.12 ± 264.68	992.18 ± 231.69	959.28 ± 284.41	0.418	0.677
Total cholesterol (mmol/L)	4.57 ± 1.00	4.66 ± 0.81	4.52 ± 1.11	0.470	0.641
Triglyceride (mmol/L)	1.64 (1.08, 2.82)	1.47 (1.20, 2.66)	1.67 (1.04, 3.61)	−0.263	0.793
iPTH (pg/ml)	258.00 (100.23, 409.00)	258.50 (96.10, 442.00)	258.00 (152.50, 376.00)	−0.162	0.872
Calcium‐phosphorus product	3.79 ± 0.92	3.51 ± 0.94	3.96 ± 0.89	−1.686	0.098
Bicarbonate (mmol/L)	25.06 ± 2.61	25.27 ± 2.49	24.95 ± 2.70	0.420	0.676
Serum ferritin (ug/L)	290.20 (253.93, 400.45)	257.80 (243.10, 281.00)	328.05 (274.65, 436.00)	−2.971	0.003
Transferrin saturation (%)	27.71 ± 6.81	24.05 ± 4.97	29.77 ± 6.90	−3.087	0.003
CRP (mg/L)	1.95 (0.58, 5.84)	1.06 (0.54, 6.85)	2.94 (1.22, 5.66)	−1.152	0.249

*Notes*: Data: n (%) for categorical; mean ± SD for normal; median (IQR) for non‑normal. Comparisons: chi‑square, t‑test, or Mann‑Whitney U as appropriate. *p* < 0.05 (two‑tailed) significant.

Abbreviations: BMI, body mass index; CRP, C‐reactive protein; ESRD, end‐stage renal disease; iPTH, intact parathyroid hormone; LDH, lactate dehydrogenase; MCI, mild cognitive impairment; NCI, non‐cognitive impairment.

### 3.2 Neuropsychology and Magnetic Resonance Data Analysis

**FIGURE 2:**
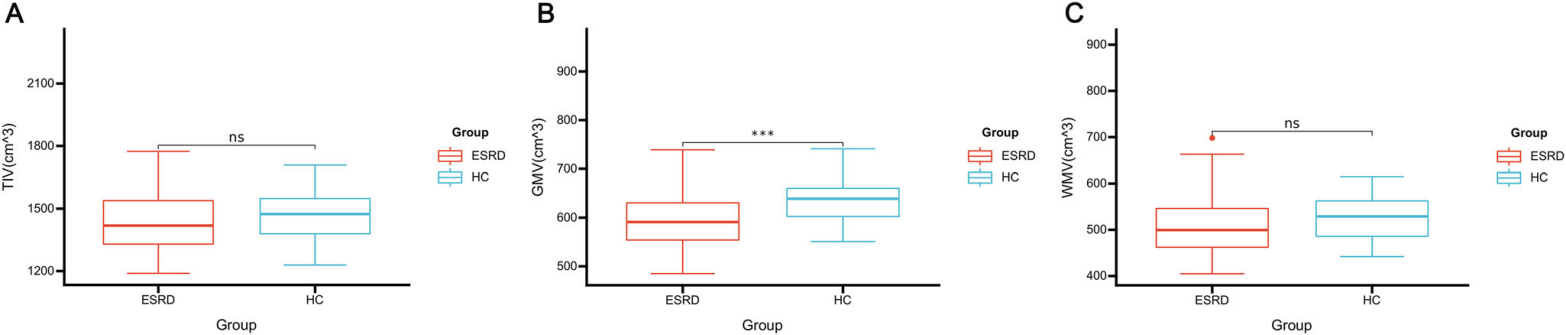
Comparisons of global brain volumetric metrics between nondialysis ESRD patients and HCs. Box‐and‐whisker plots show the differences in (A) TIV, (B) GMV, and (C) WMV between the ESRD group (*n* = 50) and the HC group (*n* = 48). Between‐group comparisons were performed using independent‐samples *t*‐test for normally distributed data. (A) TIV: *p* = 0.287, Cohen's *d* = −0.217; (B) GMV: *p* < 0.001, Cohen's *d* = −0.780; and (C) WMV: *p* = 0.138, Cohen's *d* = −0.302. Boxes represent the interquartile range, the internal horizontal line indicates the median, and whiskers extend to the minimum and maximum values. ESRD, end‐stage renal disease; GMV, gray matter volume; HC, healthy control; ns, not significant; TIV, total intracranial volume; WMV, white matter volume. ***p < 0.001.

The findings of the study indicated that no statistical differences regarding age, gender, and years of education were detected when comparing ESRD patients with HCs (all *p* > 0.05). However, the scores on both MMSE and MoCA assessments for ESRD patients were markedly lower than those recorded for the normal control group (all *p* < 0.001). Details are shown in Table 2. Regarding brain volume, the total cerebral GMV of ESRD patients was considerably less than that of the HC group (*p* < 0.001), while TIV and WMV exhibited no significant differences between the two groups (*p* > 0.05) (Figure 2).

**TABLE 2:** Comparisons of neuropsychological scores and brain volumetric metrics between nondialysis ESRD patients and healthy controls.

variables	ESRD (*n* = 50)	HC (*n* = 48)	t/χ^2^/z	*p* value
Age (years)	50.74 ± 8.07	49.46 ± 8.18	0.781	0.437
Female [n(%)]	31 (62.0)	33 (68.7)	0.492	0.483
Years of education (years)	8 (6, 9)	9 (8, 9)	−0.884	0.399
MMSE (scores)	27 (22, 29)	29 (28, 30)	−4.216	< 0.001
MoCA (scores)	24 (17, 27)	27 (25, 29)	−3.967	< 0.001
TIV (cm^3^)	1439.44 ± 146.17	1469.25 ± 128.22	−1.072	0.287
GMV (cm^3^)	595.12 ± 57.12	635.06 ± 44.29	−3.858	< 0.001
WMV (cm^3^)	508.58 ± 64.48	525.52 ± 45.77	−1.494	0.138
Hippocampus volume (cm^3^)	6.21 ± 0.81	6.78 ± 0.52	−4.119	< 0.001
Thalamus volume (cm^3^)	5.60 ± 1.17	6.08 ± 0.92	−2.266	0.026
Prefrontal cortex volume (cm^3^)	101.25 ± 11.38	108.73 ± 8.91	−3.610	< 0.001
Anterior cingulate volume (cm^3^)	8.47 ± 1.05	9.29 ± 1.02	−3.934	< 0.001

*Notes*: Data presentation and group comparisons followed the same procedures as described in Table 1. A two‐tailed *p* < 0.05 was considered statistically significant.

Abbreviations: ESRD, end‐stage renal disease; GMV, gray matter volume; HC, healthy control; MMSE, mini‐mental state examination; MoCA, Montreal cognitive assessment; TIV, total intracranial volume; WMV, white matter volume.

### 3.3 Correlation Analysis

**FIGURE 3:**
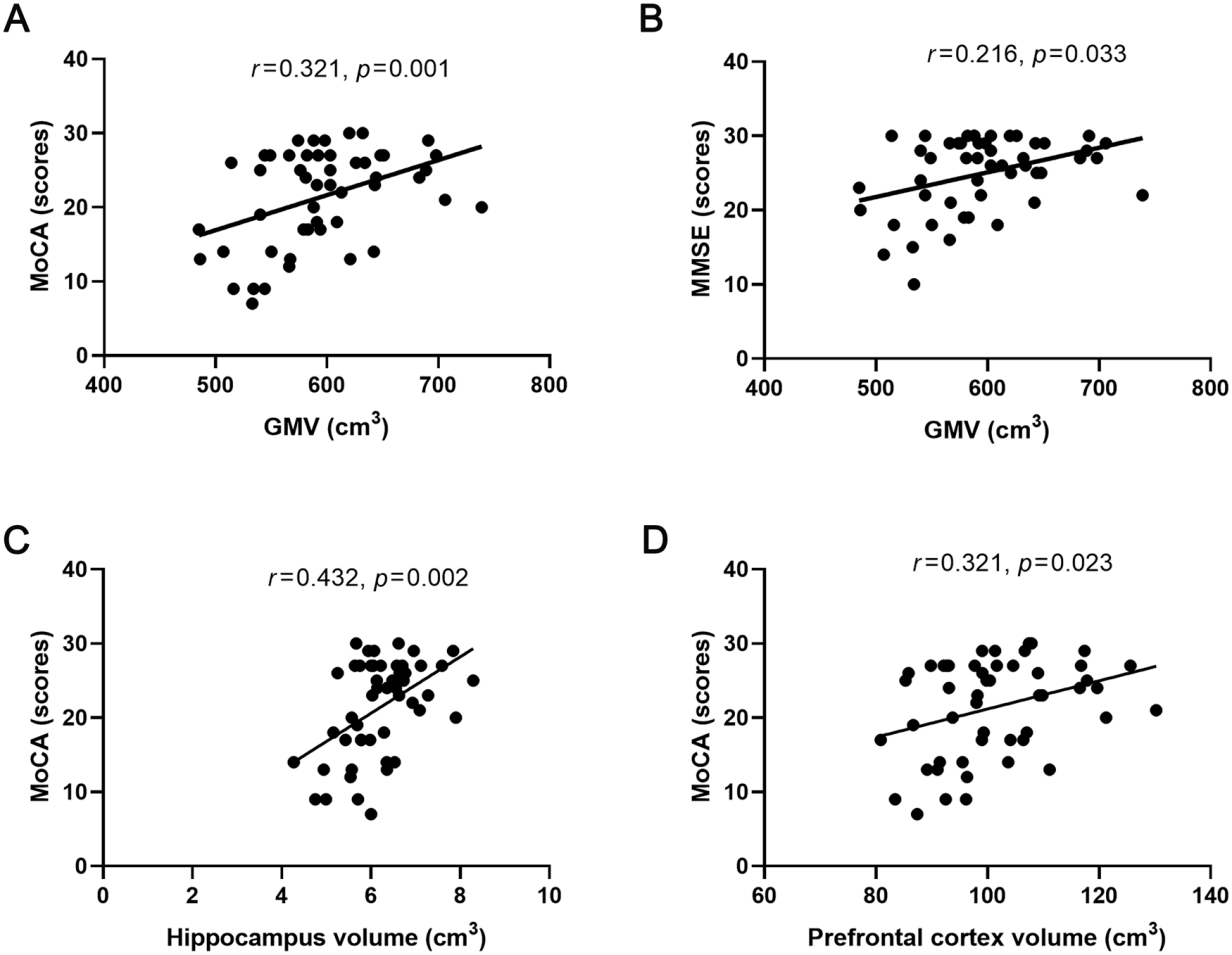
Correlations between gray matter volumes and cognitive scores in nondialysis ESRD patients (*n* = 50). Scatter plots with fitted regression lines illustrate the positive associations between (A) GMV and MoCA scores, (B) total GMV and MMSE scores, (C) hippocampal volume and MoCA scores, and (D) prefrontal cortex volume and MoCA scores. Statistical significance was determined via Spearman rank correlation analysis. (A) *r* = 0.321, *p* = 0.001; (B) *r* = 0.216, *p* = 0.033; (C) *r* = 0.435, *p* = 0.002; (D) *r* = 0.321, *p* = 0.023. ESRD, end‐stage renal disease; GMV, gray matter volume; MMSE, mini‐mental state examination; MoCA, Montreal Cognitive Assessment.

To investigate how changes in brain structure relate to cognitive abilities, a Spearman correlation analysis was conducted between total cerebral GMV and cognitive scores. The findings indicated a significant positive correlation between GMV and MMSE and MoCA scores in ESRD patients (*r* = 0.216, *p* = 0.033; *r* = 0.321, *p* = 0.001). As shown in Figure 3A–B, a decrease in total cerebral GMV is significantly associated with the decline in global cognitive function in ESRD patients.

**FIGURE 4:**
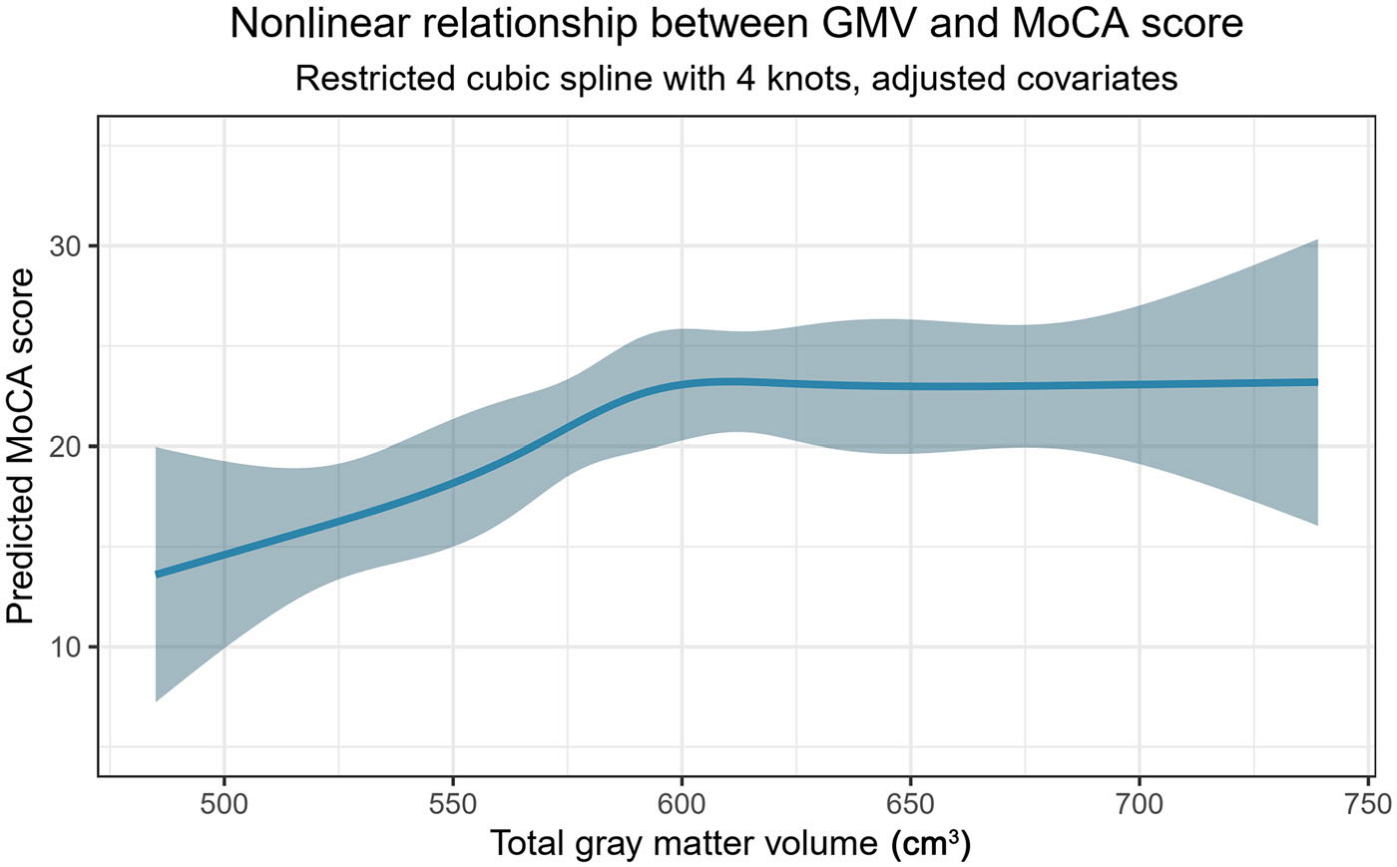
Nonlinear dose–response relationship between total GMV and MoCA scores in nondialysis ESRD patients (*n* = 50). RCS regression with 4 knots placed at the 5th, 35th, 65th, and 95th percentiles of the GMV distribution was used to model the association, with adjustment for age, years of education, serum ferritin, and transferrin saturation. The solid line represents predicted MoCA scores, and the shaded area indicates the 95% confidence interval. The formal test for nonlinearity yielded *F* = 1.46, df = 3, *p* = 0.2403, indicating no statistically significant nonlinear association. Visually, the curve shows a plateau phase when GMV is above approximately 610.7 cm^3^, followed by a steep decline in MoCA scores with further GMV reduction. ESRD, end‐stage renal disease; df, degrees of freedom; GMV, gray matter volume; MoCA, Montreal cognitive assessment; RCS, restricted cubic spline.

RCS analysis was performed to investigate the association between global GMV and MoCA scores, with adjustments for age, years of education, ferritin, and transferrin saturation. Figure 4 shows that predicted MoCA scores remained relatively stable despite mild cerebral atrophy when the total GMV was above approximately 610.7 cm^3^. Once total GMV fell below this threshold, MoCA scores decreased sharply in tandem with progressive gray matter loss. Although the fitted curve visually exhibited this segmented trend, the formal test for nonlinearity yielded a non‐significant result (*F* = 1.46, df = 3, *p* for nonlinearity = 0.2403), indicating that this apparent inflection point was not statistically validated in the present cohort.

### 3.4 The Relationship Between Gray Matter Volume and Clinical Indicators

We conducted a multiple linear regression analysis to determine the independent factors that influence total cerebral GMV in patients with ESRD. Findings from the univariate regression analysis suggested that smoking history, LDH levels, and ferritin levels might be associated with cerebral atrophy in ESRD patients (shown in Table 3). After adjusting for potential confounding factors including smoking history, serum LDH levels and CRP level, serum ferritin levels were found to be significantly and independently negatively correlated with GMV (Standardized *β* = ‐0.352, 95% CI: −0.344 to −0.057, *p* = 0.007). The results suggest that disorders related to iron metabolism could act as a significant and independent risk factor for cerebral gray matter structural damage in the complex metabolic environment of ESRD.

**TABLE 3:** Univariate and multivariate linear regression analyses of factors associated with total GMV in nondialysis ESRD patients (*n* = 50).

Variables	Univariate analysis		Multivariate analysis	
	Standardized β (95% CI)	*p* value	Standardized β (95% CI)	*p* value
Age (years)	−0.160 (−0.447, 0.126)	0.266		
Education (years)	0.149 (−0.138, 0.436)	0.302		
BMI (kg/m2)	−0.122 (−0.410, 0.166)	0.397		
Smoking history [n (%)]	0.320 (0.045, 0.595)	0.024	0.235 (−0.024, 0.494)	0.074
Drinking history [n (%)]	0.175 (−0.111, 0.461)	0.224		
Iron supplementation history [n (%)]	−0.149 (−0.436, 0.138)	0.301		
Diabetes [n (%)]	0.119 (−0.169, 0.407)	0.412		
Systolic blood pressure (mmHg)	−0.042 (−0.332, 0.248)	0.772		
Diastolic blood pressure (mmHg)	0.123 (−0.165, 0.411)	0.396		
White blood cell count (10^9^/L)	0.207 (−0.077, 0.490)	0.150		
Hemoglobin (g/L)	0.228 (−0.054, 0.511)	0.111		
Albumin (g/L)	0.023 (−0.268, 0.313)	0.876		
Alkaline phosphatase (U/L)	−0.102 (−0.391, 0.186)	0.479		
LDH (U/L)	0.305 (0.028, 0.581)	0.031	0.243 (−0.015, 0.502)	0.064
Serum creatinine (umol/L)	0.253 (−0.006, 0.115)	0.076		
Total cholesterol (mmol/L)	−0.215 (−0.499, 0.068)	0.133		
Triglyceride (mmol/L)	−0.274 (−0.553, 0.005)	0.054		
iPTH (pg/ml)	0.159 (−0.128, 0.445)	0.270		
Calcium‐phosphorus product	−0.041 (−0.331, 0.249)	0.779		
Bicarbonate (mmol/L)	−0.186 (−0.471, 0.099)	0.195		
Serum ferritin (ug/L)	−0.376 (−0.645, −0.107)	0.007	−0.352 (−0.604, −0.100)	0.007
Transferrin saturation (%)	0.069 (−0.220, 0.359)	0.632		
CRP (mg/L)	−0.147 (−0.434, 0.140)	0.308	−0.133 (−0.384, 0.118)	0.291

*Notes*: Total GMV is the dependent variable. Variables with univariate *p* < 0.05 or clinically relevant were included in the multivariate model. Results are presented as standardized regression coefficients (β) with 95% confidence intervals (CI) and corresponding *p* values.

Abbreviations: BMI, body mass index; CI, confidence interval; CRP, C‐reactive protein; ESRD, end‐stage renal disease; GMV, gray matter volume; iPTH, intact parathyroid hormone; LDH, lactate dehydrogenase.

### 3.5 Region‐Specific Gray Matter Volume Analysis

ESRD patients showed a notable decrease in GMVs across all 4 selected ROIs in comparison to HCs, with the most significant atrophy detected in the hippocampus (*p* < 0.001, Table 2). To further identify specific brain structures susceptible to iron‐related structural injury, we analyzed the associations between serum ferritin levels and GMV of 4 targeted regions. Spearman correlation analysis revealed a significant negative correlation between serum ferritin levels and the GMV in the hippocampus (*r* = −0.312, *p* = 0.028) and the anterior cingulate cortex (*r* = −0.333, *p* = 0.018). No statistically significant association was detected between ferritin and GMV of the thalamus (*r* = 0.004, *p = *0.977) or the prefrontal cortex (*r* = −0.278, *p* = 0.050). Consistent with the global GMV findings, the GMVs of hippocampus and prefrontal cortex were all positively correlated with MoCA scores (both *p* < 0.05) (shown in Figure 3C–D). These results imply that the hippocampus may be the core neuroanatomical region linking iron overload to cognitive impairment in ESRD patients.

### 3.6 Mediating Effect of Hippocampal Volume

**FIGURE 5:**
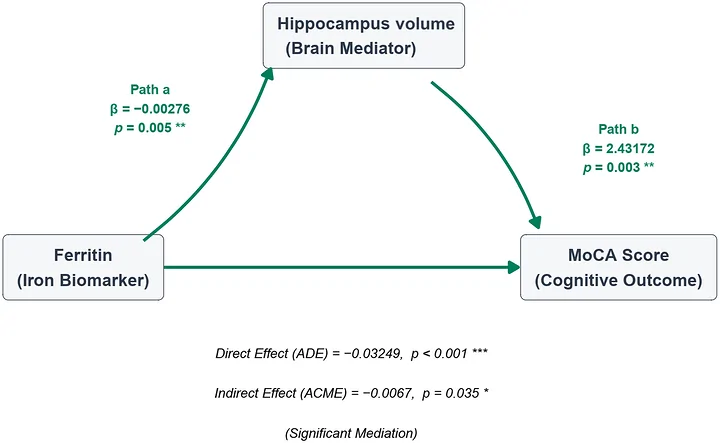
Mediation model of hippocampal volume as a mediator between serum ferritin and cognitive impairment in nondialysis ESRD patients (*n* = 50). Path diagram demonstrates the mediating role of hippocampal volume in the association between serum ferritin (independent variable) and MoCA scores (dependent variable). Path coefficients are presented as standardized β values with corresponding *p* values. Path a: effect of ferritin on hippocampal volume (*β* = −0.00276, *p* = 0.005). Path b: effect of hippocampal volume on MoCA scores (*β* = 2.43172, *p* = 0.003). ADE: *β* = −0.03249, *p* < 0.001. ACME: *β* = −0.0067, *p* = 0.035. Mediation analysis was performed using the quasi‐Bayesian Monte Carlo method with 5000 bootstrap resamples. The model confirms a significant partial mediating effect of hippocampal atrophy, explaining approximately 17.1% of the total effect of ferritin on cognitive decline. ACME, average causal mediation effect; ADE, average direct effect; ESRD, end‐stage renal disease; MoCA, Montreal cognitive assessment. *p < 0.05, **p < 0.01, ***p < 0.001.

As shown in the mediation pathway diagram (Figure 5), Path a analysis indicated a significant correlation between elevated serum ferritin levels and a decrease in hippocampal volume (*β* = −0.00276, *p* = 0.005). Path b analysis showed that larger hippocampal volume was independently associated with higher MoCA scores (*β* = 2.43172, *p* = 0.003). The indirect effect of ferritin on MoCA scores via hippocampal volume was statistically significant (average causal mediation effect, ACME = −0.0067, 95% CI: −0.0132 to −0.0008, *p* = 0.035). Following the adjustment for hippocampal volume, the analysis revealed that the direct impact of ferritin levels on MoCA scores remained significant (average direct effect, ADE = −0.03249, *p* < 0.001). These results indicate that hippocampal atrophy is a significant partial mediator in the association between elevated serum ferritin levels and cognitive impairment, accounting for approximately 17.1% of the overall impact of ferritin on cognitive function.

## 4 Discussion

This research utilized VBM to verify that patients with ESRD exhibit a reduction in global cerebral GMV, and the degree of atrophy is significantly correlated with the decline in cognitive function scores. After multiple confounding variables were accounted for, increased serum ferritin concentrations emerged as a distinct risk factor associated with the loss of GMV. Our results also suggest that maintaining relatively low serum ferritin levels within the clinical safe range may be a protective factor for preserving gray matter structure and cognitive function in ESRD patients. This finding not only provides a neuroanatomical basis for ESRD‐related cognitive impairment but, more importantly, establishes abnormal iron metabolism as a potential modifiable risk factor, thereby offering a novel direction for the exploration of targeted neuroprotective strategies.

Notably, the plateauing pattern observed in our RCS curve aligns with the well‐established cognitive reserve theory, which posits that the brain can maintain functional performance over a considerable range of structural loss until a critical atrophy threshold is breached (Li et al. 2026). In our ESRD cohort, MoCA scores increased substantially with GMV in the lower‐volume range, but this positive association attenuated when GMV exceeded a certain level, implying that additional gray matter beyond this point confers limited incremental cognitive benefit. Nevertheless, the lack of statistical significance in the nonlinearity test (*p* = 0.2403) prevents us from definitively concluding a fixed threshold. The limited sample size we had could partially account for the non‐significant nonlinearity, as it did not provide enough statistical power to identify subtle threshold inflection points. Larger cohorts are required to further verify whether a cognitive reserve threshold exists in this patient population.

The primary constituents of cerebral gray matter include neuronal cell bodies, dendrites, and glial cells, which act as the critical substrate for the execution of advanced cognitive functions. The maintenance of GMV relies on the structural integrity and normal metabolic function of neurons (Cobia et al. 2024). Previous studies have consistently reported reductions in GMV in medial temporal lobe structures (e.g., the hippocampus and entorhinal cortex) in patients with MCI (Luo et al. 2022). The present study demonstrated a significant atrophy of global cerebral GMV, in ESRD patients, with the degree of atrophy positively correlated with MMSE and MoCA scores, which is consistent with the findings of prior research. The neural mechanisms underlying this correlation may be multifaceted. First, the long‐term accumulation of uremic toxins (e.g., indoxyl sulfate) can directly damage neuronal cells, interfere with energy metabolism, and induce synaptic dysfunction. In addition, uremic toxin accumulation can alter the structure and diffusion characteristics of the extracellular space in the brain, impair the clearance of brain metabolic waste, and disrupt the homeostasis of the brain microenvironment, which is also a potentially important mechanism behind structural and functional damage among ESRD individuals (Watanabe et al. 2021; Viggiano et al. 2020; de Donato et al. 2022). As reported by Buonincontri et al. (2025), impaired kidney function can compress brain extracellular space (ECS) even in non‑dialysis patients, potentially driven by circulating TGF‑β, neuroinflammation, and brain extracellular‑matrix remodeling (Buonincontri et al. 2025). Compressed ECS impairs glymphatic perivascular clearance and slows the elimination of interstitial uremic metabolites and labile iron. For our ESRD cohort, ECS contraction may act as a synergistic pathological factor, amplifying iron‑mediated oxidative neurotoxicity and aggravating gray‑matter atrophy and cognitive decline. Nevertheless, our study did not acquire diffusion tensor image analysis along the perivascular space (DTI‐ALPS) data to quantify ECS directly. Future multimodal MRI combining brain volumetry and DTI‐ALPS measurements is needed to validate this mechanistic pathway. Second, pro‐inflammatory cytokines (e.g., interleukin‐6 [IL‐6], tumor necrosis factor‐α [TNF‐α]) released during chronic inflammation may impair neurons by activating microglia and promoting neuroinflammation (Yang et al. 2025). Thus, cerebral gray matter atrophy in ESRD patients is not attributable to a singular cause; rather, it results from the combined effects of various metabolic disorders, which can be clinically detected as volume loss via MRI and accompanied by assessable cognitive decline.

Investigating the factors influencing brain structural changes in ESRD patients is crucial for identifying high‐risk populations and discovering intervention targets. Through multiple regression analysis, the present study found that serum ferritin is an independent factor affecting GMV, with elevated levels significantly correlated with reduced GMV. Iron is an essential trace element for normal brain physiological activities, participating in myelination, neurotransmitter synthesis, and energy metabolism (Muñoz and Humeres 2012). However, excess iron can induce oxidative stress, generating reactive oxygen species via the Fenton reaction, resulting in neuronal damage and ultimately causing cellular injury and death (Muñoz and Humeres 2012). In ESRD patients, excessive iron load is common due to the administration of erythropoietin (EPO), repeated blood transfusions, and intravenous iron supplementation. This study is the first to directly link serum ferritin—a marker reflecting systemic iron storage levels—to brain structural damage in the ESRD population. Consistent with the global GMV findings, our region‐specific analysis further revealed heterogeneous vulnerability of different brain regions to iron overload in ESRD patients. The hippocampus exhibited the most significant atrophy and the strongest correlation with serum ferritin levels, suggesting it is the most susceptible brain structure to iron‐related neurotoxicity. This region‐specific pattern is supported by prior evidence: the hippocampus has high metabolic activity and abundant neuronal populations, making it particularly vulnerable to oxidative damage induced by iron overload (Zhang et al. 2024). Excess iron can accumulate in hippocampal neurons, trigger ferroptosis, and impair synaptic plasticity, ultimately leading to volume atrophy and memory dysfunction (Tian et al. 2023; Zeng et al. 2023). The sparing of the thalamus and prefrontal cortex in our ferritin correlation analyses does not imply that these regions are unaffected in ESRD, but rather suggests that their volume loss may be driven by other uremic factors (e.g., hypertension, uremic toxins) rather than iron overload per se. The potential mechanisms may include the following: First, excess iron may cross the compromised blood‐brain barrier and deposit in gray matter nuclei such as the basal ganglia and substantia nigra, triggering regional oxidative damage (Li et al. 2015). Second, oxidative stress catalyzed by iron can impair vascular endothelial function, exacerbate cerebral microangiopathy, and indirectly cause neuronal ischemia (Gao et al. 2024). The continued presence of this relationship after accounting for the systemic inflammatory marker CRP supports the independent neurotoxic effect of iron overload. However, residual confounding by other inflammatory factors (e.g., IL‐6, TNF‐α) cannot be ruled out. Third, as an acute‐phase protein, elevated ferritin levels often reflect a state of systemic chronic inflammation, which can further activate microglia, promote pro‐inflammatory substance secretion, and accelerate neuronal degeneration (Wang et al. 2023).

Notably, our mediation analysis further confirmed that hippocampal atrophy partially mediates the adverse effect of elevated ferritin on cognitive function. This finding establishes a complete neuroanatomical pathway: excess systemic iron storage in ESRD patients leads to hippocampal structural atrophy, which in turn contributes to cognitive decline. The partial mediation pattern indicates that, in addition to hippocampal atrophy, iron overload may impair cognitive function through other pathways, such as white matter injury, neuroinflammation, and synergistic toxicity with uremic toxins (Liu et al. 2025; Andrews et al. 2025), which warrant further investigation in future studies. From a clinical perspective, the mediating role of the hippocampus suggests that targeted iron management to protect hippocampal structure may be a potential strategy to delay cognitive decline in ESRD patients.

In clinical practice, iron supplementation is the standard treatment for renal anemia in ESRD patients (including both nondialysis and dialysis populations). However, our results suggest that it is necessary to balance the benefit of anemia correction with the potential neurotoxicity caused by iron overload. Currently, there is no evidence to support maintaining ferritin levels below physiological values. Excessively low ferritin will affect erythropoiesis and aggravate anemia, which is also detrimental to cognitive function and brain health. Existing clinical guidelines have established target ferritin ranges for ESRD patients based on cardiovascular and hematological safety (Jalal et al. 2026). This study provides preliminary evidence from the perspective of neurological safety, suggesting that persistent hyperferritinemia caused by excessive iron supplementation should be avoided. Future prospective studies are needed to explore the optimal ferritin target interval that takes both hematopoietic efficacy and neuroprotection into account.

While this study has important translational implications for clinical iron management, it also presents several notable limitations. First, this study reveals the correlation between ferritin and cerebral GMV but cannot establish a causal relationship. Further verification is required through prospective cohort studies. Second, the relatively small sample size, consisting exclusively of ESRD patients, could restrict the applicability of the findings. Third, no blood biochemical tests (serum ferritin, CRP, etc.) were performed in the healthy control group. This major limitation precludes assessment of whether the ferritin‐GMV association is specific to the uremic milieu or also present in the general population. Future studies should include control subjects with complete blood panels to address this question. Fourth, although multiple clinical indicators were adjusted for, some potential confounding factors—such as medication history (particularly the use of EPO) and nutritional status—were not included in the analysis, which may have exerted a certain impact on the results. Fifth, this study only included nondialysis ESRD patients, so the results cannot be directly generalized to dialysis populations. Factors such as dialysis vintage, dialysis modality, and acute fluid shifts during dialysis may interact with iron metabolism and affect brain structure. Sixth, although we performed ROI analyses based on a priori hypotheses derived from prior literature, the number of regions examined was limited to four, and we did not apply correction for multiple comparisons across all 116 AAL parcellations due to the exploratory nature of this analysis and the modest sample size. Future studies can further verify the generalizability of our conclusions in dialysis cohorts.

## 5 Conclusions

In summary, this study confirmed through VBM analysis that ESRD patients exhibit brain structural alterations characterized by reduced global GMV, and that these structural changes are significantly correlated with cognitive impairment. In addition, the research revealed that increased serum ferritin concentrations in ESRD patients constitute an independent risk factor for GMV reduction, with the hippocampus and anterior cingulate cortex being particularly vulnerable. Crucially, hippocampal volume loss partially mediates the ferritin–cognitive impairment pathway, providing a novel mechanistic insight. The GMV–cognition association exhibits a significant overall positive trend with a suggestive plateau at higher GMV volumes, hinting at a potential reserve mechanism; however, formal threshold identification was constrained by sample size. Future longitudinal research involving larger samples is essential to confirm the exact shape of this relationship and to determine whether ferritin‐targeted interventions could mitigate neurocognitive decline in this vulnerable population.

## Author Contributions

**Jingjing Si**: conceptualization, formal analysis, investigation, methodology, writing – original draft, writing – review and editing. **Minghua Zhu**: investigation, methodology, project administration, resources. **Mingzhe Dai**: conceptualization, supervision, validation, writing – original draft, writing – review and editing. All authors contributed to the final manuscript. All authors read and approved the final manuscript.

## Funding

The authors have nothing to report.

## Conflicts of Interest

The authors declare no conflicts of interest.

## Data Availability

Data are available upon reasonable request from the corresponding author.
